# Cuprate-like electronic structures in infinite-layer nickelates with substantial hole dopings

**DOI:** 10.1093/nsr/nwae194

**Published:** 2024-06-04

**Authors:** Xiang Ding, Yu Fan, Xiaoxiao Wang, Chihao Li, Zhitong An, Jiahao Ye, Shenglin Tang, Minyinan Lei, Xingtian Sun, Nan Guo, Zhihui Chen, Suppanut Sangphet, Yilin Wang, Haichao Xu, Rui Peng, Donglai Feng

**Affiliations:** Advanced Materials Laboratory, State Key Laboratory of Surface Physics, and Department of Physics, Fudan University, Shanghai 200433, China; Advanced Materials Laboratory, State Key Laboratory of Surface Physics, and Department of Physics, Fudan University, Shanghai 200433, China; Advanced Materials Laboratory, State Key Laboratory of Surface Physics, and Department of Physics, Fudan University, Shanghai 200433, China; Advanced Materials Laboratory, State Key Laboratory of Surface Physics, and Department of Physics, Fudan University, Shanghai 200433, China; Advanced Materials Laboratory, State Key Laboratory of Surface Physics, and Department of Physics, Fudan University, Shanghai 200433, China; Advanced Materials Laboratory, State Key Laboratory of Surface Physics, and Department of Physics, Fudan University, Shanghai 200433, China; Advanced Materials Laboratory, State Key Laboratory of Surface Physics, and Department of Physics, Fudan University, Shanghai 200433, China; Advanced Materials Laboratory, State Key Laboratory of Surface Physics, and Department of Physics, Fudan University, Shanghai 200433, China; Advanced Materials Laboratory, State Key Laboratory of Surface Physics, and Department of Physics, Fudan University, Shanghai 200433, China; Advanced Materials Laboratory, State Key Laboratory of Surface Physics, and Department of Physics, Fudan University, Shanghai 200433, China; Advanced Materials Laboratory, State Key Laboratory of Surface Physics, and Department of Physics, Fudan University, Shanghai 200433, China; School of Emerging Technology, University of Science and Technology of China, Hefei 230026, China; New Cornerstone Science Laboratory, University of Science and Technology of China, Hefei 230026, China; Advanced Materials Laboratory, State Key Laboratory of Surface Physics, and Department of Physics, Fudan University, Shanghai 200433, China; Shanghai Research Center for Quantum Sciences, Shanghai 201315, China; Advanced Materials Laboratory, State Key Laboratory of Surface Physics, and Department of Physics, Fudan University, Shanghai 200433, China; Shanghai Research Center for Quantum Sciences, Shanghai 201315, China; National Synchrotron Radiation Laboratory and School of Nuclear Science and Technology, University of Science and Technology of China, Hefei 230026, China; Advanced Materials Laboratory, State Key Laboratory of Surface Physics, and Department of Physics, Fudan University, Shanghai 200433, China; School of Emerging Technology, University of Science and Technology of China, Hefei 230026, China; New Cornerstone Science Laboratory, University of Science and Technology of China, Hefei 230026, China

**Keywords:** unconventional superconductivity, nickelate superconductors, oxide MBE, ARPES, electronic structure, superconducting phase diagram

## Abstract

Superconducting infinite-layer (IL) nickelates offer a new platform for investigating the long-standing problem of high-temperature superconductivity. Many models were proposed to understand the superconducting mechanism of nickelates based on the calculated electronic structure, and the multiple Fermi surfaces and multiple orbitals involved create complications and controversial conclusions. Over the past five years, the lack of direct measurements of the electronic structure has hindered the understanding of nickelate superconductors. Here we fill this gap by directly resolving the electronic structures of the parent compound LaNiO_2_ and superconducting La_0.8_Ca_0.2_NiO_2_ using angle-resolved photoemission spectroscopy. We find that their Fermi surfaces consist of a quasi-2D hole pocket and a 3D electron pocket at the Brillouin zone corner, whose volumes change upon Ca doping. The Fermi surface topology and band dispersion of the hole pocket closely resemble those observed in hole-doped cuprates. However, the cuprate-like band exhibits significantly higher hole doping in superconducting La_0.8_Ca_0.2_NiO_2_ compared to superconducting cuprates, highlighting the disparities in the electronic states of the superconducting phase. Our observations highlight the novel aspects of the IL nickelates, and pave the way toward the microscopic understanding of the IL nickelate family and its superconductivity.

## Teaser

The long sought electronic structure of infinite-layer nickelate superconductors is resolved for the first time, representing a milestone toward the microscopic understanding of its superconductivity.

## INTRODUCTION

Following the discovery of high-temperature superconductivity in cuprates [1], it was suggested that superconductivity in nickelates could be realized if the common Ni 3d^8^ state could be reduced to 3d^9^ [2–6]. Indeed, superconductivity was discovered in (Nd, Sr)NiO_2_ thin films [7] after the apical oxygens in (Nd, Sr)NiO_3_ films were removed by reaction with CaH_2_ powders. Subsequently, superconductivity was achieved in related compounds such as (La, Sr)NiO_2_ [8,9], (La, Ca)NiO_2_ [10], (Pr, Sr)NiO_2_ [11] and (Nd, Eu)NiO_2_ [12]. However, high-quality infinite-layer (IL) nickelate superconductors are difficult to fabricate [13–15], and the surface of IL nickelate superconductors usually becomes disordered in the reduction process [16,17], which prevents the reliable measurement of its electronic structure by angle-resolved photoemission spectroscopy (ARPES) or scanning tunneling microscopy (STM).

Various theoretical models on the superconductivity of IL nickelates are based on combinations of different Ni/*RE* (rare earth) orbitals and Fermi surface topologies. Consequently, distinct superconducting mechanisms could be reached. For instance, Kitatini *et al.* propose that *RE*NiO_2_ can be described by the one-band Hubbard model with an Ni-3*d_x^2^-y^2^_* orbital akin to cuprates, based on which superconducting transition temperature can be estimated [18]. However, others suggested that Ni-3*d_xy_* or Ni-3*d*_3_*_z^2-r^^2^_* orbitals and Hund's coupling should be included, potentially yielding a high-spin *S* = 1 state in superconducting nickelates [19,20]. Additionally, the presence of conduction electrons (including various *RE-d* orbitals and interstitial *s* orbitals) and their contributions to superconductivity further complicate the understanding [21–28]. Depending on different hybridization and doping levels, various pairing symmetries distinct from hole-doped cuprates were predicted [29–31].

Since accurate knowledge of the low-energy electronic structure is critical for modeling the IL nickelates, many fundamental issues need to be pinned down, such as the Fermi surface topology, hole concentration, the orbital characters of bands, and the participation of *RE-5d* or interstitial *s* orbitals in the low-energy electronic structure. Particularly, a key question is whether the electronic structure resembles those of cuprates. However, due to strong electron correlations, an accurate band calculation for IL nickelates is still challenging, thus direct experimental studies are demanded.

## RESULTS

### Single-crystalline IL surface

**Figure 1.**
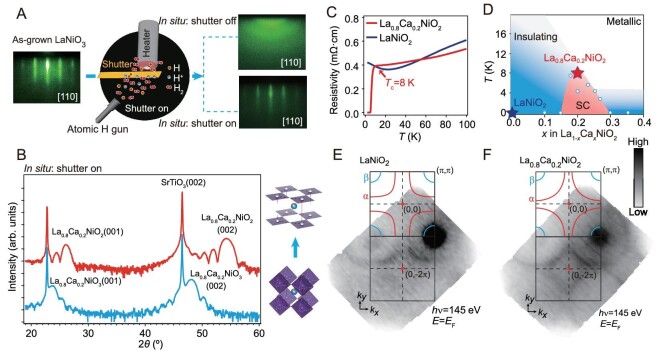
*In-situ* reduction and optimization to get atomically flat and clean surfaces for ARPES measurements. (A) Evolution of RHEED image along the [110] azimuth after *in-situ* reduction. The RHEED pattern is single-crystalline (poly-crystalline) when the shutter is on (off) after reduction. The shutter was designed to screen the by-product H^+^ generated with atomic H. (B) XRD *θ*-2*θ* scans of the perovskite 25 uc La_0_*_._*_8_Ca_0_*_._*_2_NiO_3_/SrTiO_3_ and *in-situ* reduced IL La_0_*_._*_8_Ca_0_*_._*_2_NiO_2_/SrTiO_3_. (C) Temperature-dependent resistivity curves of the LaNiO_2_/SrTiO_3_ and La_0_*_._*_8_Ca_0_*_._*_2_NiO_2_/SrTiO_3_ samples in this study. (D) Superconducting *T*_c_ vs. Ca doping level plot in the phase diagram of (La, Ca)NiO_2_ adapted from ref. [10]. The open circles represent data points reported in ref. [10], while the filled stars illustrate the data obtained from our samples. (E, F) Photoemission intensity map of 21 uc LaNiO_2_/SrTiO_3_ and 25 uc La_0_*_._*_8_Ca_0_*_._*_2_NiO_2_/SrTiO_3_ at *E*_F_. The integration is over the energy window of *E*_F_ ± 0.1 eV. The red rounded rectangle and the blue small pockets are denoted as *α* and 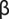 pockets, respectively.

Reliable measurements of the electronic structure of IL nickelates require high-quality stoichiometric *RE*NiO_3_ perovskite films, sufficient *in-situ* reduction, and most critically, single-crystalline IL surfaces. Achieving single-crystalline IL surfaces poses a significant challenge, as it requires a delicate balance in the strength of the reduction conditions: strong enough to facilitate a topotactic transition to the IL phase, yet mild enough to prevent damage to the crystalline surface, which is essential for surface-sensitive techniques like ARPES. To address this challenge, we have performed *in-situ* reduction and systematically optimized the reducing conditions. We have grown LaNiO_3_ and La_0_*_._*_8_Ca_0_*_._*_2_NiO_3_ thin films on SrTiO_3_ (001) substrates using oxide molecular beam epitaxy with an atomic-layer-by-layer growth method (see Materials and Methods in the Supplementary  Data), and then reduced them *in situ* with atomic hydrogen. As depicted in Fig. 1A, we have used a shutter to prevent direct H atom bombardment on the sample surface, which effectively avoids disorder formation during the violent topotactic reduction process. In this way, the reflection high-energy electron diffraction (RHEED) pattern of the reduced films shows sharp streaks from the surfaces (Fig. 1A), and atomic force microscopy (AFM) shows terraces with unit-cell step height (Fig. S4), indicating single-crystalline and atomically flat sample surfaces. *Ex-situ* X-ray diffraction (XRD) measurements were performed on the same samples after ARPES measurements (Fig. 1B and Fig. S3). The positions of the diffraction peaks shift to higher values compared to those of the perovskite phase (Fig. 1B), and are consistent with those of (La, Ca)NiO_2_ [10]. The fringes accompanying the diffraction peaks observed in La_0_*_._*_8_Ca_0_*_._*_2_NiO_2_ (Fig. 1B) and LaNiO_2_ (Fig. S3A) are comparable to, if not more pronounced than, the previous reports [8–10,17]. The conversion efficiency from perovskite to IL phase is among the highest in the literature [17] (Supplementary Data). These results demonstrate the acquisition of the IL phase with superior surface quality. According to the resistivity measurements (Fig. 1C), 21 unit cell (uc) LaNiO_2_/SrTiO_3_ shows a weakly insulating behavior below 25 K, while 25 uc La_0_*_._*_8_Ca_0_*_._*_2_NiO_2_/SrTiO_3_ shows a superconducting transition at 8 K. These behaviors are in line with the reported phase diagram of (La, Ca)NiO_2_ (Fig. 1D, [10]). ARPES measurements on these samples show clear Fermi surfaces (Fig. 1E–F).

### Cuprate-like band dispersion

**Figure 2.**
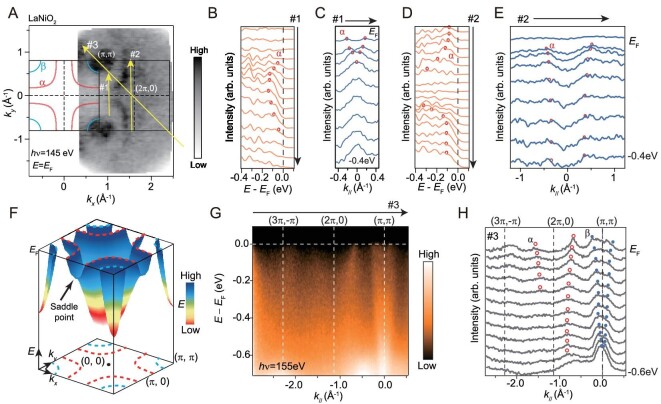
Cuprate-like low-energy electronic structure of LaNiO_2_. (A) Photoemission intensity map of 21 uc LaNiO_2_/SrTiO_3_ at *E*_F_. The integration is over the energy window of *E*_F_ ± 0.1 eV. The Fermi surfaces of a cuprate-like hole pocket *α* and an electron pocket 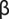 are illustrated. (B) Energy distribution curves (EDCs) along the momentum cut #1. (C) Momentum distribution curves (MDCs) along the momentum cut #1. (D) The same as panel B, but along cut #2. (E) The same as panel C, but along cut #2. (F) Schematic dispersion of *α* and 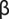 bands. The saddle point of the α band is indicated. (G) Photoemission intensity along cut #3. (H) MDCs along cut #3. The circle markers track the local maxima/shoulders to demonstrate the dispersion of *α* band (red circles) and 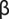 band (blue circles).

Figure 2 shows the detailed electronic structure of LaNiO_2_ measured by ARPES. The *α* band resembles the low-energy Zhang-Rice singlet of cuprates in terms of both Fermi surface shape and band dispersion [32]. It forms a large rounded square pocket centered at (*π, π*) with parallel sectors around (*π*, 0) (Fig. 2A). Note that the spectral weight intensity is higher in the second Brillouin zone (BZ) (Figs 1E and F, and 2A), a phenomenon commonly observed in ARPES studies of cuprates and consistent with the photoemission matrix-element of 3*d_x^2^*-y*^2^_* orbitals, and our polarization-dependent ARPES measurements also support its *d_x^2^*-y*^2^_* character (as shown in Fig. S5 of the Supplementary Data). The dispersion of *α* band exhibits a shallow electron-like dispersion along cut #1 in Fig. 2B and C. Along cut #2, the dispersion of the *α* band is steep near the zone center (Fig. 2E) and it flattens towards lower binding energy near (0, *π*) (Fig. 2D). These demonstrate a saddle-point dispersion in the (0, *π*) region (Fig. 2F), akin to the dispersion in the anti-nodal region of cuprates [32]. The saddle-point dispersion is also observed in La_0_*_._*_8_Ca_0_*_._*_2_NiO_2_ (Fig. S6), highlighting its similarity to cuprates. On the other hand, there is an electron-like band centered at (*π, π*) (Fig. 2G–H), which is absent in cuprates. Note that the quasiparticle weight is weak but discernible here, manifested by the sharper peaks in the momentum distribution curves near the Fermi level (Fig. 2H) and by the reduced band velocity as the energy approaches the Fermi energy (Fig. 2G). The spectral width of the α band broadens as the energy moves away from the Fermi level, and the dispersion of the *α* band becomes steeper at binding energies beyond 0.2 eV, similar to the steep ‘waterfall’ dispersion observed in the cuprates [33].

### Distinct hole-doping phase diagram

**Figure 3.**
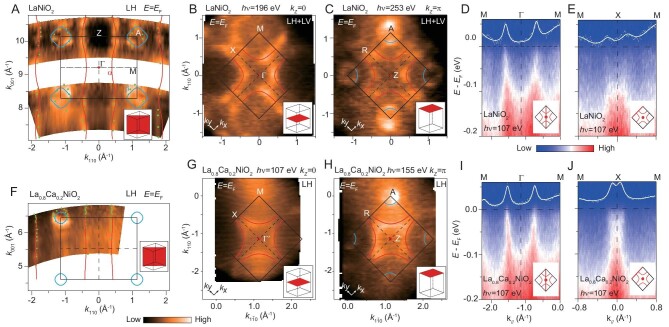
Doping dependence of the 3D electronic structure. (A) Photon-energy dependent photoemission intensity map of LaNiO_2_/SrTiO_3_ at *E*_F_ in the Γ-M-A-Z plane. (B) Photoemission intensity map at *E*_F_ measured at the Γ-M-X plane (*k_z_* = 0). (C) Same as panel B but measured at the Z-A-R plane (*k_z_* = *π*). The linear horizontal (LH) and linear vertical (LV) polarizations of photons are indicated, corresponding to the photoemission geometry of *π*-polarization and σ-polarization, respectively. (D–E) Photoemission spectra along the M-Γ-M direction (D) and M-X-M direction (E) of LaNiO_2_/SrTiO_3_, measured using 107 eV photons (*k_z_* = 0). The MDCs at *E*_F_ were overlaid to show the Fermi crossings. (F–J) Same as panels A–E but measured on La_0_*_._*_8_Ca_0_*_._*_2_NiO_2_/SrTiO_3_.

To reveal the 3D electronic structure of IL nickelates, we further conducted photon energy-dependent ARPES measurements on the films. As shown in Fig. 3A and F, the *α* band shows weak dispersion along *k_z_*, demonstrating its quasi-2D character, whereas the 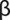 band is 3D and only appears at the A point. In the Γ-M-X plane, the *α* Fermi surface forms a large hole pocket centered at M (Fig. 3B and G), consistent with theoretical calculations [34–36] (Fig. S8). The electron pocket with dominant La-5*d*_3_*_z^2^*-r*^2^_* character predicted at Γ is absent in the experiment (Fig. 3B and G). In Fig. 3C and H, the circular pocket formed by the 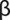 band is identified around the A point. It is noteworthy that the Fermi surface of the *α* band in the Z-A-R plane roughly matches that in the Γ-M-X plane, consistent with its quasi-2D character. This is different from the prediction by density functional theory (DFT) calculations, where the hole pockets of the *α* band around A expand and transform into an electron pocket around Z in the Z-A-R plane [34].

**Figure 4.**
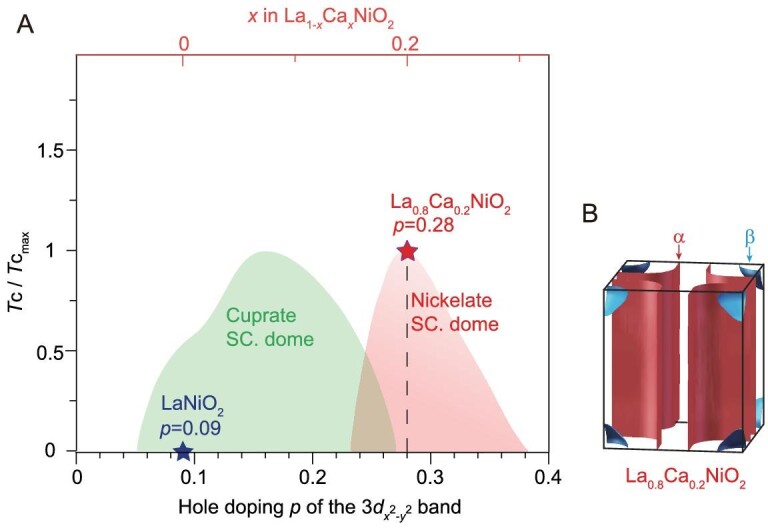
Substantial hole doping in the quasi-2D *α* band. (A) Hole doping at the 3*d_x^2^-y^2^_* orbital plotted on the cuprate phase diagram. (B) Sketch of the measured 3D Fermi surfaces observed in La_0_*_._*_8_Ca_0_*_._*_2_NiO_2_.

As a function of the hole doping in the Zhang-Rice singlet band, cuprate superconductors show a generic phase diagram across various families of materials [37]. Here we compare the doping of the cuprate-like *α* band of Ni-3*d_x^2^*-y*^2^_* character in IL nickelates with the general phase diagram of cuprates. According to the Luttinger theorem and the measured Fermi surface volume, the quasi-2D Fermi surface of the *d_x^2^*-y*^2^_* band possesses 1.09 holes in LaNiO_2_ (see Supplementary Data for details), indicating an excess of 0.09 holes relative to the 3*d*^9^ electronic configuration, far from the half-filled Mott insulator. Upon Ca substitution, the 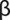 pocket also shrinks (Fig. S7), and the Fermi crossings of the *α* band shift away from the M point along both the M-Γ-M direction (Fig. 3D and I) and M-X-M direction (Fig. 3E and J), indicating an increase of the hole pocket size. The estimated hole concentration is 1.28 for the *α* pocket of La_0_*_._*_8_Ca_0_*_._*_2_NiO_2_. Therefore, despite the resemblance in the band dispersion, the cuprate-like *α* band in optimally doped La_0_*_._*_8_Ca_0_*_._*_2_NiO_2_ possesses an ultra-high doping level of 28%, placing it in the over-doped and non-superconducting regime of the cuprates [37]. As illustrated in the phase diagram (Fig. 4A), the superconducting dome of IL nickelates shows up at a higher doping regime of the *d_x^2^*-y*^2^_* band than that of cuprates, which highlights the intriguing differences between the nickelate and cuprates.

## DISCUSSION

The quasi-2D *α* band dominated by the Ni-3*d_x^2^*-y*^2^_* orbital and the 3D 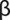 band around A qualitatively agree with the previous DFT results, where the orbital characters are predicted to be Ni-3*d_x^2^*-y*^2^_* for *α* band, and the mixing of La-5*d_xy_*, Ni-3*d_zx_*/*d_yz_* and interstitial-*s* for 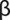 band, respectively ([34], Fig. S8). Nonetheless, some discrepancies with the calculated electronic structures are evident. Specifically, there is no electron pocket around Γ in LaNiO_2_ (Fig. 4B), and the 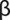 electron Fermi pocket around the A point shows a slightly smaller size and a shallower band bottom than the calculations (Fig. S7j). These observations consistently suggest that the self-doping effect is weaker than the calculation prediction. The 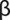 and *α* bands show different *E*_F_ shifts upon doping (Fig. S7), indicating a non-rigid-band behavior. These discrepancies may be attributed to electron correlation effects overlooked in previous calculations, which potentially alters the dispersion [38]. Our ARPES data thus give a benchmark for further improving theoretical calculations.

In comparison to hole-doped cuprates, although the *α* band in IL nickelates generally captures the dispersion characteristics of the Zhang-Rice singlet band in cuprates, their superconducting doping ranges are far apart. As shown in Fig. 4A, the doping level of the *α* band in the parent compound LaNiO_2_ already falls within the superconducting regime of cuprates, whereas the superconducting La_0_*_._*_8_Ca_0_*_._*_2_NiO_2_ resides at the highly overdoped and non-superconducting regime of cuprates. The distinct superconducting hole-doping regimes of cuprates and IL nickelates may be rooted in the different nature of their electronic states. In cuprates, holes are predominantly doped into oxygen, and the states near the Fermi level primarily consist of oxygen ligand states. However, due to the large charge transfer gap in IL nickelates [21,39], the contribution of oxygen *p* states is significantly less in IL nickelates compared to cuprates, and the *α* bands are predominantly dominated by the *d_x^2^*-y*^2^_* orbital (Fig. S8). Furthermore, the 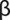 band persists in the optimally doped superconducting La_0_*_._*_8_Ca_0_*_._*_2_NiO_2_, suggesting the multiband nature of nickelate superconductors, distinct from that of cuprate superconductors.

To summarize, by reaching an unprecedented surface quality, we experimentally revealed the low-energy electronic structure of IL nickelates. Our ARPES measurements have revealed a large hole pocket *α* that bears resemblance to the Zhang-Rice singlet Fermi surface and dispersion in cuprates, and holes could be effectively introduced by Ca doping. The weak but finite self-doping effect, together with the highly hole-doped superconducting state, differs from the electronic structure of cuprates, posing constraints to theories. These findings clarify the fundamental issues regarding the electronic structure of IL nickelates, and pave the way toward understanding the superconductivity mechanism in IL nickelates. The observed large hole-doping level in the superconducting La_0.8_Ca_0.2_NiO_2_ is intriguing. It encourages the study of other IL nickelate systems, especially (Nd, Eu)NiO_2_, which exhibits an additional doping level difference. The method developed for obtaining the single-crystalline surface of IL nickelates opens avenues for further surface-sensitive experimental studies on this family of compounds.

## MATERIALS AND METHODS

### Thin film growth

Perovskite (La, Ca)NiO_3_ thin films were grown on TiO_2_-terminated SrTiO_3_(001) substrates by oxide molecular-beam epitaxy. A layer-by-layer growth mode was used, in which the A site element (La, Ca) and B site element (Ni) were deposited alternatively, while La and Ca were co-deposited to get uniform doping. The flux of each element was measured by quartz crystal microbalance (QCM), and then calibrated by Rutherford backscattering spectrometry (RBS) measurements. X-ray reflection (XRR) measurements were performed to further calibrate the absolute thickness of the films. XRD measurements were performed to optimize the growth conditions. After optimization, LaNiO_3_ and Ca-doped LaNiO_3_ were grown at 580°C under an ozone pressure of 5 × 10^−6^ mbar and 1.5 × 10^−5^ mbar, respectively. The 2D character of the RHEED pattern was maintained during growth, indicating a single-crystalline and 2D sample surface. The doping level calibrated by RBS was then further checked by X-ray photoemission on the samples after ARPES studies.

### *In-situ* reduction

After growth, the precursor thin films were transferred *in situ* to the pulsed laser deposition (PLD) chamber for reduction. Our PLD system is integrated with an atomic hydrogen gun, which generates atomic hydrogen by dissociating H_2_ gas through plasma. IL LaNiO_2_ and La_0.8_Ca_0.2_NiO_2_ thin films were obtained by annealing perovskite precursors in an atomic hydrogen environment for 1–2 hours at 340°C, with a ramp rate of 15°C/min. During the reduction process, the H_2_ gas flow rate was fixed at ∼3 sccm, and the chamber pressure was around 1.0 × 10^−5^ mbar. A metal shutter was used to prevent surface crystal structure degradation caused by exposure to H^+^ (Fig. 1A). Under the optimized conditions, perovskite nickelates were transformed into IL nickelates, as confirmed by the XRD pattern (see Fig. 1B and Fig. S3a). Meanwhile, the fully strained feature (Fig. S3b) and the terraced surfaces were maintained in IL samples (Fig. S4).

### ARPES measurements

All the ARPES experiments were performed at the Shanghai Synchrotron Radiation Facility (SSRF). All samples were reduced *in situ* and then transferred to beamline by vacuum suitcases and measured under an ultra-high vacuum lower than 7 × 10^−11^ mbar. The soft X-ray (SX) ARPES data and the complementary vacuum ultraviolet (VUV) ARPES data were collected at beamline 09U and beamline 03U, respectively. In VUV-ARPES experiments, we set the energy resolution power to 3000 for higher photon flux, which gives a typical energy resolution of ∼40 meV at 145 eV photon energy. The estimated energy resolution of SX-ARPES is ∼100 meV at ∼250 eV, and ∼200 meV at ∼400 eV. The angle resolution is 0.1°.

More details on materials and methods can be found in the Supplementary Data [40–43].

## Supplementary Material

nwae194_Supplemental_File
