# Supplementary material for: Cuprate-like electronic structures in infinite-layer nickelates with substantial hole dopings
Source: Natl Sci Rev. 2024 Jun 4;11(8):nwae194. doi: 10.1093/nsr/nwae194 (PMC11242455; doi:10.1093/nsr/nwae194)
Supplement: nwae194_Supplemental_File [file nwae194_supplemental_file.docx]

Supplementary Materials for

**Cuprate-like Electronic Structures in Infinite-Layer Nickelates with Substantial Hole Dopings**

Xiang Ding^1^†, Yu Fan^1^†, Xiaoxiao Wang^1^, Chihao Li^1^, Zhitong An^1^, Jiahao Ye^1^, Shenglin Tang^1^, Minyinan Lei^1^, Xingtian Sun^1^, Nan Guo^1^, Zhihui Chen^1^, Suppanut Sangphet^1^, Yilin Wang^2,3^, Haichao Xu^1,4^*, Rui Peng^1,4^*, Donglai Feng^5,1,2,3^*

Corresponding authors: [xuhaichao@fudan.edu.cn,](about:blank) [pengrui@fudan.edu.cn,](mailto:pengrui@fudan.edu.cn) [dlfeng@ustc.edu.cn.](about:blank)

**The PDF file includes:**

Detailed Materials and Methods

Supplementary Text

Figs. S1 to S8

Tables S1

References [40-43]

**Detailed Materials and Methods**

Thin films growth

Perovskite (La, Ca)NiO_3_ thin films were grown on TiO_2_-terminated SrTiO_3_ (001) substrates by oxide molecular beam epitaxy (OMBE). To prevent photoemission charging effects, silver paste was applied to the substrate edge during mounting.

Each metal evaporation source in the OMBE chamber has an independent pneumatic shutter, allowing the atomic beams to be controlled individually. The films were grown using an atomic layer-by-layer method, depositing the A-site material (La/Ca) and B-site material (Ni) alternately, while Ca and La were co-deposited to achieve uniform doping. Purified ozone was used during growth, which is provided by an ozone-distilling system. Residual gas analysis (RGA) was employed to ensure consistent ozone partial pressure in the chamber throughout each growth. The flux of each element (La, Ca and Ni) was measured using quartz crystal microbalance (QCM) before and after the film growth, and calibrated using Rutherford backscattering spectrometry (RBS) (Fig. S1). Calibration samples of LaNiO_3_ and 34% Ca-doped LaNiO_3_ were grown on MgO substrates under the same growth temperature and ozone partial pressure as the corresponding samples for ARPES measurements. RBS spectra were collected at a backscattering angle of 160° with a beam energy of 2.275 MeV. The samples were rotated and tilted at a slight angle to the incident beam to prevent channeling effects in both the films and substrates. The doping level of the film calibrated by Rutherford backscattering spectrometry is further checked by X-ray photoemission on the samples. After optimization, LaNiO_3_ and Ca-doped LaNiO_3_ were grown at 580 ^◦^C under an ozone pressure of 5×10^−6^ mbar and 1.5×10^−5^ mbar, respectively, with A-site termination. After the growth of each unit cell, the films were annealed for 100 seconds. Real-time reflection high-energy electron diffraction (RHEED) indicated stable intensity oscillations during growth and preserved a two-dimensional pattern.

# *In-situ* reduction

After growth, the thin films were subsequently transferred *in-situ* to the pulsed laser deposition (PLD) chamber for reduction. Our PLD system is integrated with an atomic hydrogen gun, which generates atomic hydrogen through the dissociation of H_2_ gas via plasma.

We have conducted a series of experiments to optimize the reduction condition. During the reduction process, the hydrogen gas flow is maintained at 3 sccm to ensure the stable operation of the hydrogen source and provide sufficient atomic hydrogen for effective reduction. An insufficient flow rate would destabilize the hydrogen plasma, while an excessive flow rate would overload the pumping system. The reduction temperature and annealing time were optimized, while maintaining a constant hydrogen flow rate. Temperatures were measured using a pyrometer. As shown in Fig. S2a, (002) XRD peaks of the *in-situ* reduced samples have the same trend of reduction temperature dependence as the *ex-situ* reduced (La,Sr)NiO_3_ thin films [15]. For reduction temperatures below 330^◦^C, the broad (002) XRD peaks are located below 53.5^◦^, indicating that the samples are under-reduced (Fig. S2a). For temperatures higher than 350^◦^C, the films undergo rapid degradation (over-reduced), as demonstrated by the decreased intensity of the (002) XRD peaks. In both cases, the upturned resistivity at the lowest temperature approaches or even exceeds the room-temperature resistivity (Fig. S2b). Based on these observations, 340^◦^C is identified as an appropriate reduction temperature, as it results in the highest XRD peak intensity. Compared to the samples reduced at lower or higher temperatures, those reduced at 340^◦^C show lower resistivity at low temperatures and a lower metal-insulator transition temperature (*<*30K). Finally, a reduction duration of 1 hour was chosen as the standard condition, under which LaNiO_2_ samples exhibit the highest intensity of (002) X-ray diffraction (XRD) peaks (Fig. S2a) and the lowest resistivity (Fig. S2b). For the Ca-doped samples, it is worth noting that 1 hour is not sufficient to fully convert La_0_*_._*_8_Ca_0_*_._*_2_NiO_3_ to La_0_*_._*_8_Ca_0_*_._*_2_NiO_2_ (Fig. S2c). Extending the reaction time to 2 hours enhances the XRD diffraction peaks from the infinite-layer phase and induces superconductivity in the film. (Figs. S2c, 1C). A metal shutter was always used to prevent surface crystal structure degradation caused by exposure to H^+^ (Fig. 1a). Under the optimized conditions, perovskite nickelates were transformed into IL nickelates, as confirmed by the X-ray diffraction pattern (see Fig. 1b and Fig S3a). Meanwhile, the fully-strained feature (Fig. S3b) and the terraced surfaces were maintained in IL samples (Fig. S4).

ARPES measurements

All the ARPES experiments were performed at the Shanghai Synchrotron Radiation Facility (SSRF). All samples were reduced *in-situ* and then transferred to beamline by vacuum suitcases and measured under an ultra-high vacuum better than 7×10^−11^ mbar. The SX-ARPES data and the complementary VUV-ARPES data were collected at beamline 09U and beamline 03U, respectively. In VUV ARPES experiments, we set the energy resolution power to 3000 for higher photon flux, which gives a typical energy resolution of 40 meV at 145eV photon energy. The estimated energy resolution of SX-ARPES is 100 meV at 250 eV, and 200 meV at 400 eV. The angle resolution is 0.1◦.

DFT calculations

The DFT calculations are performed using the VASP package [40,41], with exchange-correlation functional of generalized gradient approximation (GGA) [42]. The experimental lattice parameters, *a*=*b*=3.905 Å and *c* = 3.393Å are used. The energy cutoff of the plane-wave basis is set to be 500 eV. Γ-centered 11×11×11 and 41×41×41 K-point grids are used for the charge self-consistent and Fermi surface calculations, respectively. A tight-binding (TB) Hamiltonian, consisting of La-5*d*, La-4*f* , Ni-3*d*, O-2*p* and the interstitial *s* orbital that is centered on (0,0,0.5), is constructed using the Wannier90 package [43]. The band characters are then analyzed in terms of these Wannier orbitals.

**Supplementary Text**

Characterizations of the IL films

According to XRD results, both the undoped LaNiO_3_ and doped La_0_*_._*_8_Ca_0_*_._*_2_NiO_3_ films have been successfully converted to their infinite-layer phases (Fig. 1b and Fig. S3a) after *in-situ* reduction with the shutter on. The out-of-plane lattice constants of LaNiO_2_ and doped La_0_*_._*_8_Ca_0_*_._*_2_NiO_2_ films are calculated to be 3.401 Å and 3.393 Å, respectively, based on their (002) diffraction peaks. According to the reciprocal space maps (RSM) around the (103) SrTiO_3_ diffraction peak (Fig. S3b), the in-plane lattice constants of the reduced infinite-layer thin films are fully strained to the SrTiO_3_ substrates. These results are consistent with those obtained from previous *ex-situ* CaH_2_ reduction studies [8,10].

According to Ref [17], *R*_002_ is defined as the intensity ratio of the (002) diffraction peak between the perovskite phase (*I*) and the reduced infinite-layer phase (*I*’): *R*_002_ = *I/I*^′^. This ratio is an empirical measure used to indicate the conversion efficiency from the perovskite to the infinite-layer phase (Fig. S3a). A smaller *R*_002_ value corresponds to a higher conversion efficiency from the perovskite to the infinite-layer phase. Before calculating *R*_002_, the (002) peaks of the SrTiO_3_ substrates were normalized. In our La_0_*_._*_8_Ca_0_*_._*_2_NiO_2_ film, *R*_002_ is calculated to be 5.01. Comparing this with previous results [17] our *R*_002_ value of 5.01 is among the smallest reported, indicating that our reduction method has been optimized.

Resistivity measurements of our films have been conducted after ARPES studies and XRD measurements. LaNiO_2_ exhibits metallic behavior and shows a slight upturn in resistivity below 24 K (Fig. S3c). The observed resistivity in our LaNiO_2_ samples is lower than previously reported insulating LaNiO_2_ [1], possibly due to improved sample quality. The resistivity of our LaNiO_2_ films roughly aligns with previous reports on uncapped nickelate parent compounds including PrNiO_2_ and NdNiO_2_ [2,5], while higher than that of SrTiO_3_-capped LaNiO_2_, which can exhibit a superconducting-like onset at 1 K [8]. La_0_*_._*_8_Ca_0_*_._*_2_NiO_2_ shows similar room temperature resistivity and superconducting transition temperature of 8K (Fig. S3c), higher than previously reported *T*_c_=4K [10].

AFM topography of the *in-situ* reduced LaNiO_2_ and La_0_*_._*_8_Ca_0_*_._*_2_NiO_2_ shows that step-terraced surfaces are maintained (Fig. S4). The averaged terrace height is about 3.3 Å, consistent with the XRD results mentioned earlier.

The effect of hole-doping measured by the ARPES

Comparing the *α* Fermi surfaces, the pocket size is larger in La_0_*_._*_8_Ca_0_*_._*_2_NiO_2_ than in LaNiO_2_, with a more rounded shape (Figs. S6a, S6f). The momentum distribution curves (MDCs) and energy distribution curves (EDCs) show a saddle point near (*π*,0) in La_0_*_._*_8_Ca_0_*_._*_2_NiO_2_ as well (Figs. S7g-S7j), which is raised closer to *E*_F_ when comparing Figs. S6b-S6d to Figs. S6g-S6i.

Meanwhile, the Fermi crossings of *β* only show a slight change due to the relatively large band velocity (Figs. S7).

Calculating the carrier doping from the Fermi surface volumes

We estimate the carrier density from the experimental Fermi surfaces based on the Luttinger theorem. According to the chemical composition and the charge neutral condition, the nominal carrier density of LaNiO_2_ and La_0_*_._*_8_Ca_0_*_._*_2_NiO_2_ would be 1 hole/uc and 1.2 holes/uc, respectively. If we take the quasi-2D *α* pocket to be purely 2D by approximation, the hole concentration of each unit cell for the *α* pocket is calculated by *α*_2_*_D_*=2×*A*(*α*)*/A*(*BZ*), where the *A*(*α*) and *A*(*BZ*) are the area of the round square *α* pocket and the square Brillouin zone in Figs. 1E-1F, respectively. As shown in Tab. S1, the estimated hole concentrations at *α* are 1.09 and 1.28 for LaNiO_2_ and La_0_*_._*_8_Ca_0_*_._*_2_NiO_2_, respectively. These values are used to plot the data points in Fig.3G. The electron concentration of *β* pocket (*β*3D) is estimated by its three-dimensional shape and Fermi crossings, *β*_3_*_D_*=2×*V*(*β*)*/V*(*BZ*), where the *V*(*β*) and *V*(*BZ*) are the volume of the *β* pocket and the three-dimensional Brillouin zone. The *β*3D are estimated to be 0.031 and 0.012 for LaNiO_2_ and La_0_*_._*_8_Ca_0_*_._*_2_NiO_2_, respectively.


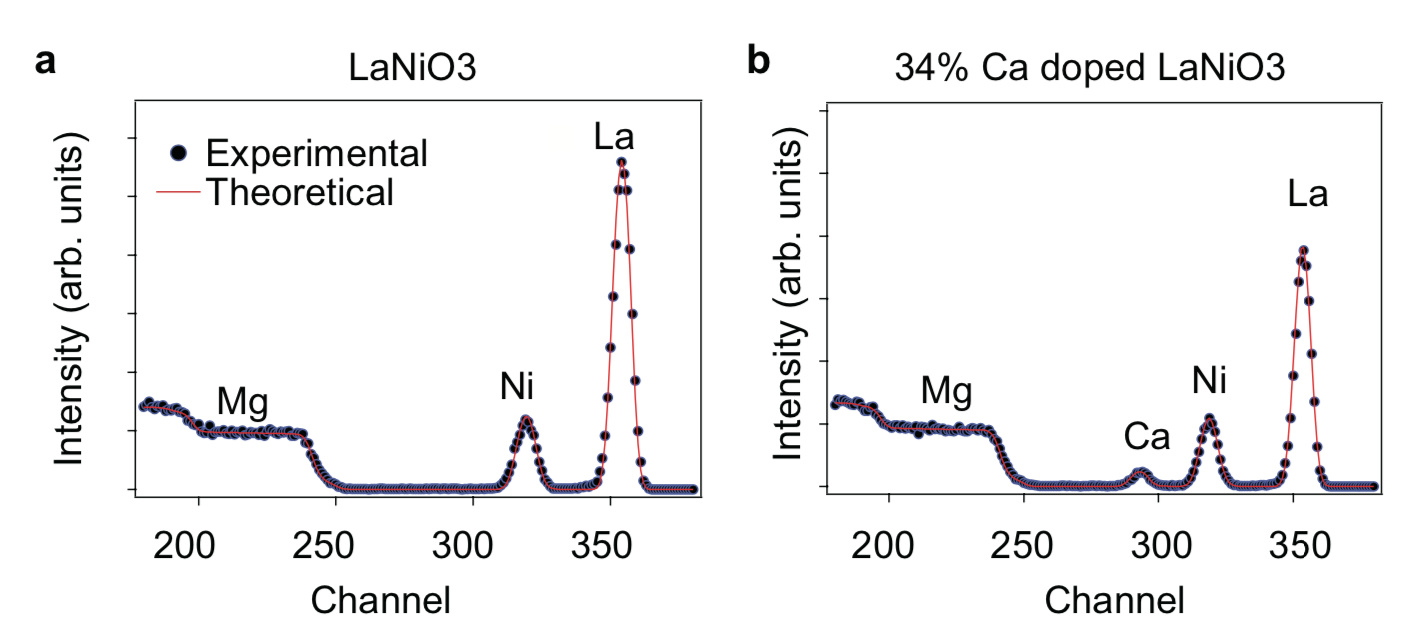


**Fig. S1.**

**Rutherford backscattering spectrometry (RBS) data for calibrating the atomic flux. a-b**, RBS spectra of the calibration samples with LaNiO_3_ and 34% Ca doped LaNiO_3_. According to the calibrated flux, we grew LaNiO_3_ and 20% Ca-doped LaNiO_3_ for ARPES measurements. The experimental data points and simulated curves are denoted by filled circles and red curves, respectively.


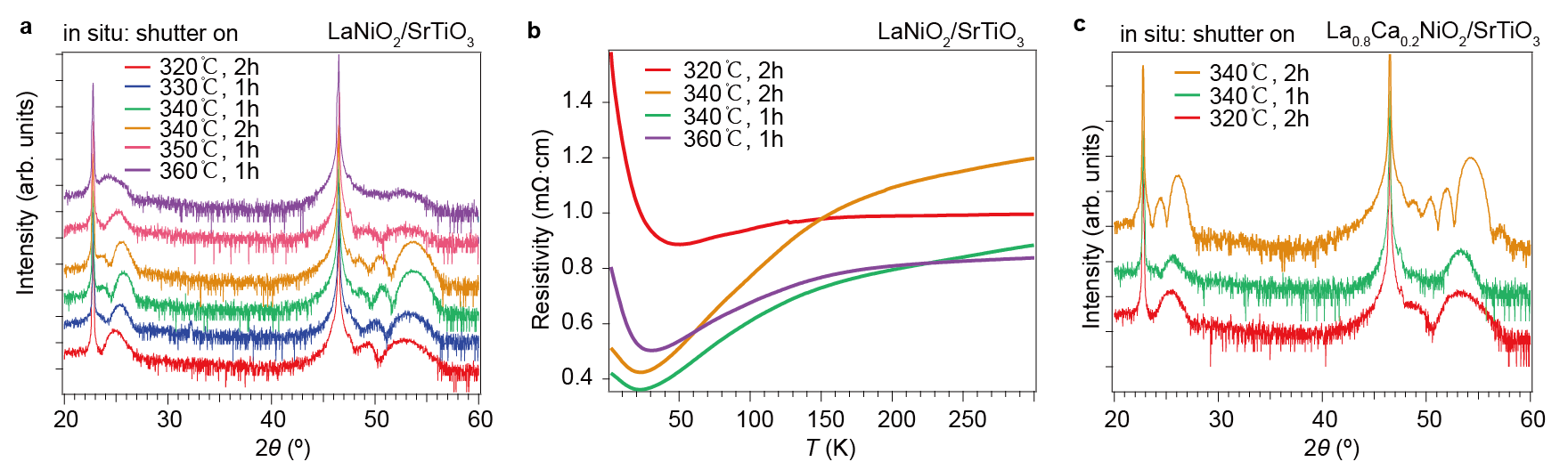


**Fig. S2.**

**Optimization of *in-situ* reduction conditions. a,** XRD of LaNiO_2_/SrTiO_3_ under different reduction temperatures and annealing times. **b**, Temperature-dependent resistivity of LaNiO_2_/SrTiO_3_ reduced under different reduction temperatures and annealing times. **c**, XRD of La_0_*_._*_8_Ca_0_*_._*_2_NiO_2_/SrTiO_3_ under different reduction temperatures and annealing times.


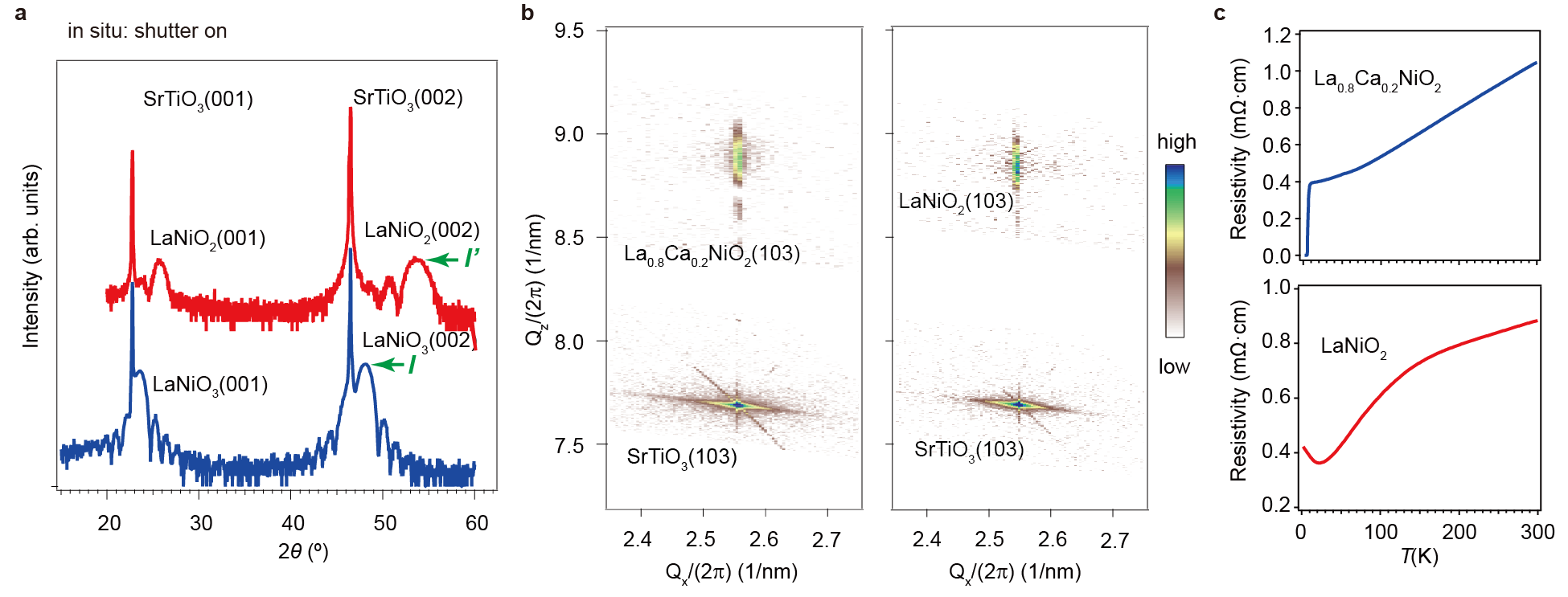


**Fig. S3.**

**XRD and resistivity measurements on the *in-situ* reduced LaNiO_2_ and La_0_*_._*_8_Ca_0_*_._*_2_NiO_2_ films.** **a**, The XRD *θ*-2*θ* scans of the perovskite 21 uc LaNiO_3_/SrTiO_3_ and *in- situ* reduced IL LaNiO_2_/SrTiO_3_. Note that the XRD curves are normalized according to the intensity of SrTiO_3_ (002) diffraction peaks before calculating the *R*_002_. **b**, Reciprocal space maps of La_0_*_._*_8_Ca_0_*_._*_2_NiO_2_ and LaNiO_2_ around the SrTiO_3_ (103) diffraction peak. **c**, Temperature-dependent resistivity of nickelates thin films.


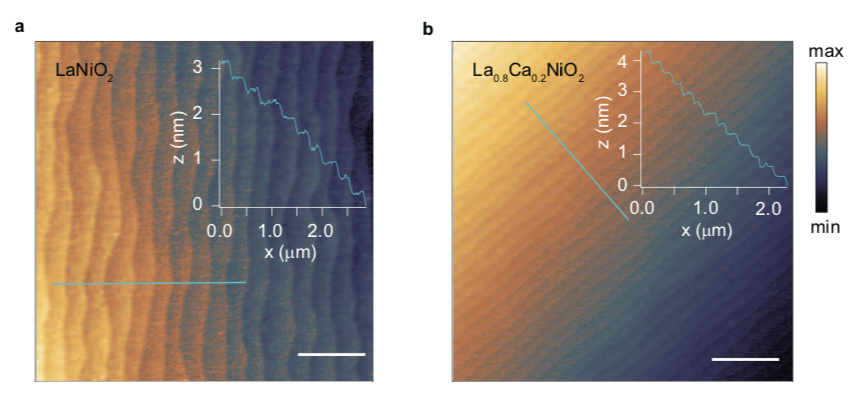


**Fig. S4.**

**AFM images of *in-situ* reduced LaNiO_2_ and La_0_*_._*_8_Ca_0_*_._*_2_NiO_2_ films. a**, AFM image of 21 uc LaNiO_2_. The root mean square roughness *R_q_* is 110±15 pm. **b**, AFM image of 25 uc La_0_*_._*_8_Ca_0_*_._*_2_NiO_2_. The *R_q_* is 125±15 pm. The inset picture shows height profiles along step terrace (cyan line). The scale bars are 1 µm.


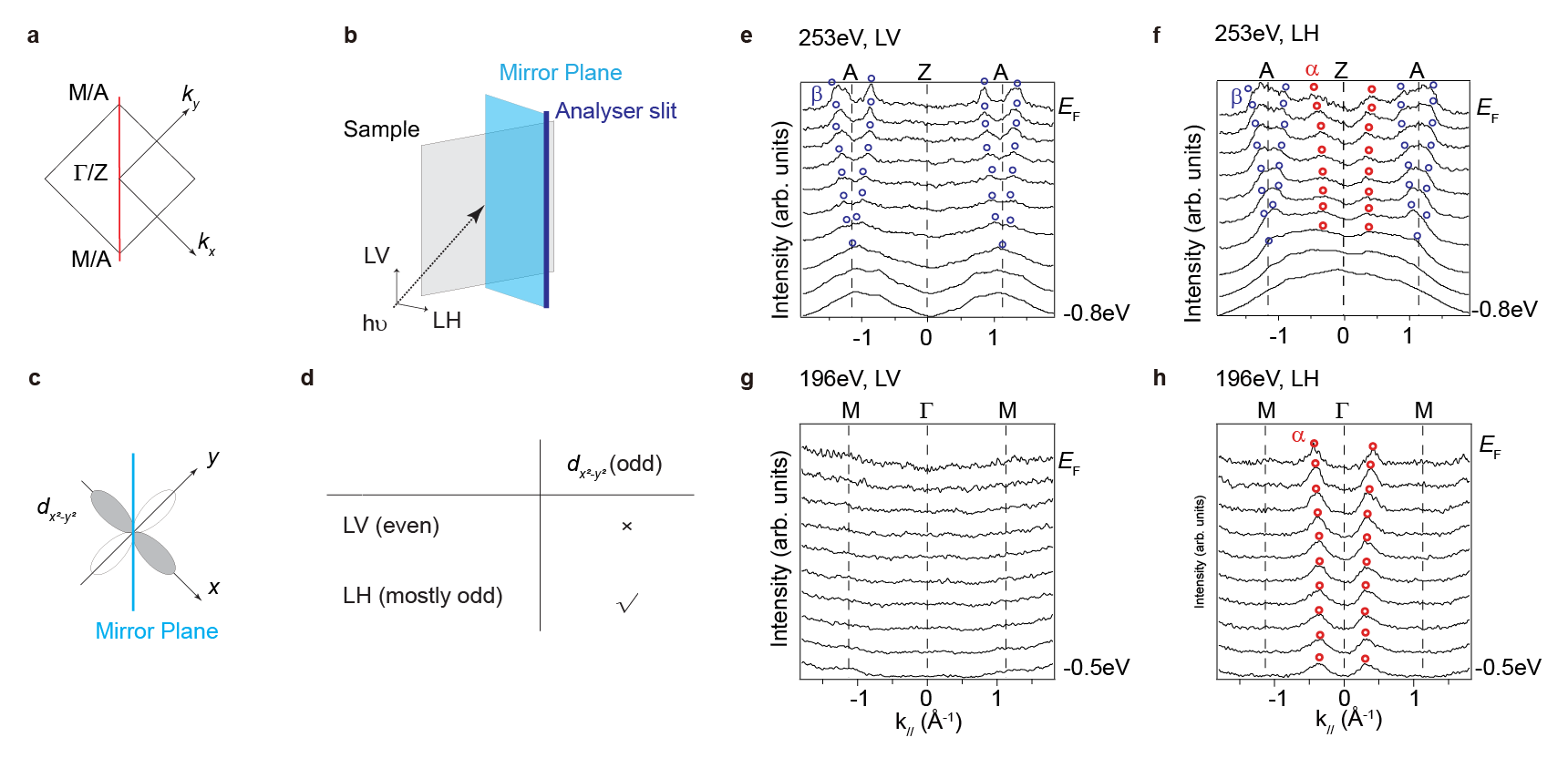


**Fig.S5.**

**Polarization-dependent behavior of α band. a**, M-Γ-M cut and A-Z-A cut in Brillouin zone. **b**, Experimental geometry used for the ARPES measurements, where the linear vertical (LV) and linear horizontal (LH) photon polarizations are illustrated. **c**, Illustration of the spatial symmetry of the 3*d_x_*_2-_*_y_*_2_ orbital with respect to the mirror plane. **d**, Symmetry of the incident photons and the electronic orbital with respect to the mirror plane, which indicate that the photoemission signal from 3*d_x_*_2-_*_y_*_2_ orbital should be suppressed at LV polarization. **e**, MDCs along A-Z-A direction measured with LV-polarized photons on LaNiO_2_/SrTiO_3_. **f**, Same as **a**, but measured with LH-polarized photons. **g-h,** Same as **e** and **f** but along the MΓM direction.


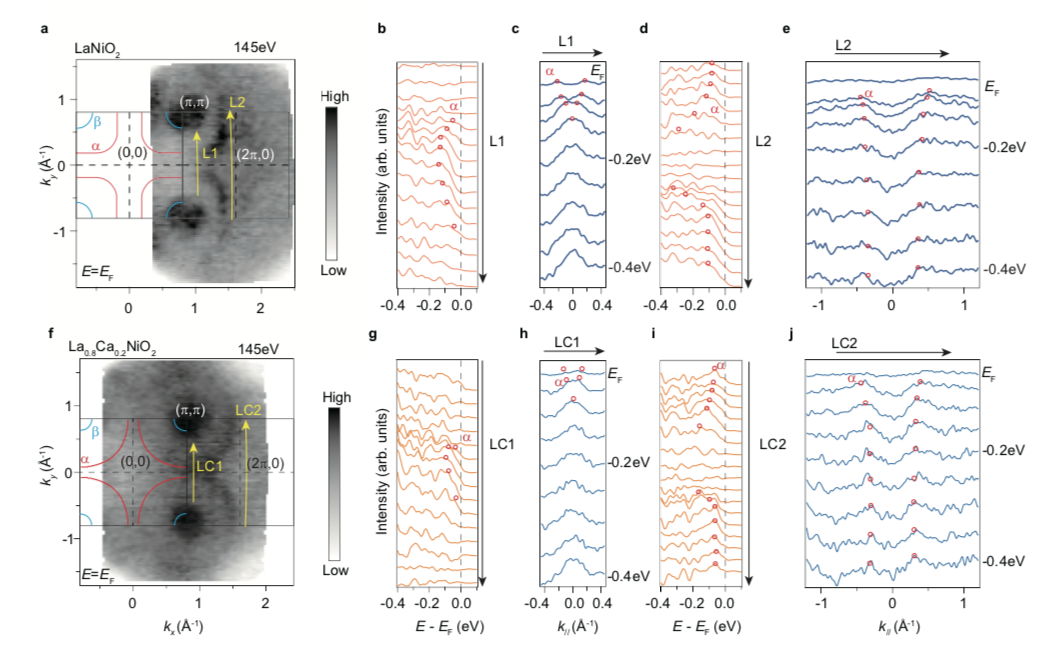


**Fig. S6.**

**Comparison of the low-energy electronic structure of La_0_*_._*_8_Ca_0_*_._*_2_NiO_2_ and LaNiO_2_**. **a**, Photoemission intensity map of 21uc LaNiO_2_/SrTiO_3_ at *E*_F_ taken with 145 eV photons. The integration is over the energy window of *E*_F_ ±0*.*1 eV. **b**, EDCs along the momentum cut L1. The circle markers track the local maxima/shoulders and demonstrate the saddle-point dispersion of *α* band around (0, *π*). **c**, MDCs along the momentum cut L1. d, EDCs along cut L2. e, MDCs along cut L2. **f-j**, the same as **a-e**, respectively, but measured on the 25uc La_0_*_._*_8_Ca_0_*_._*_2_NiO_2_/SrTiO_3._


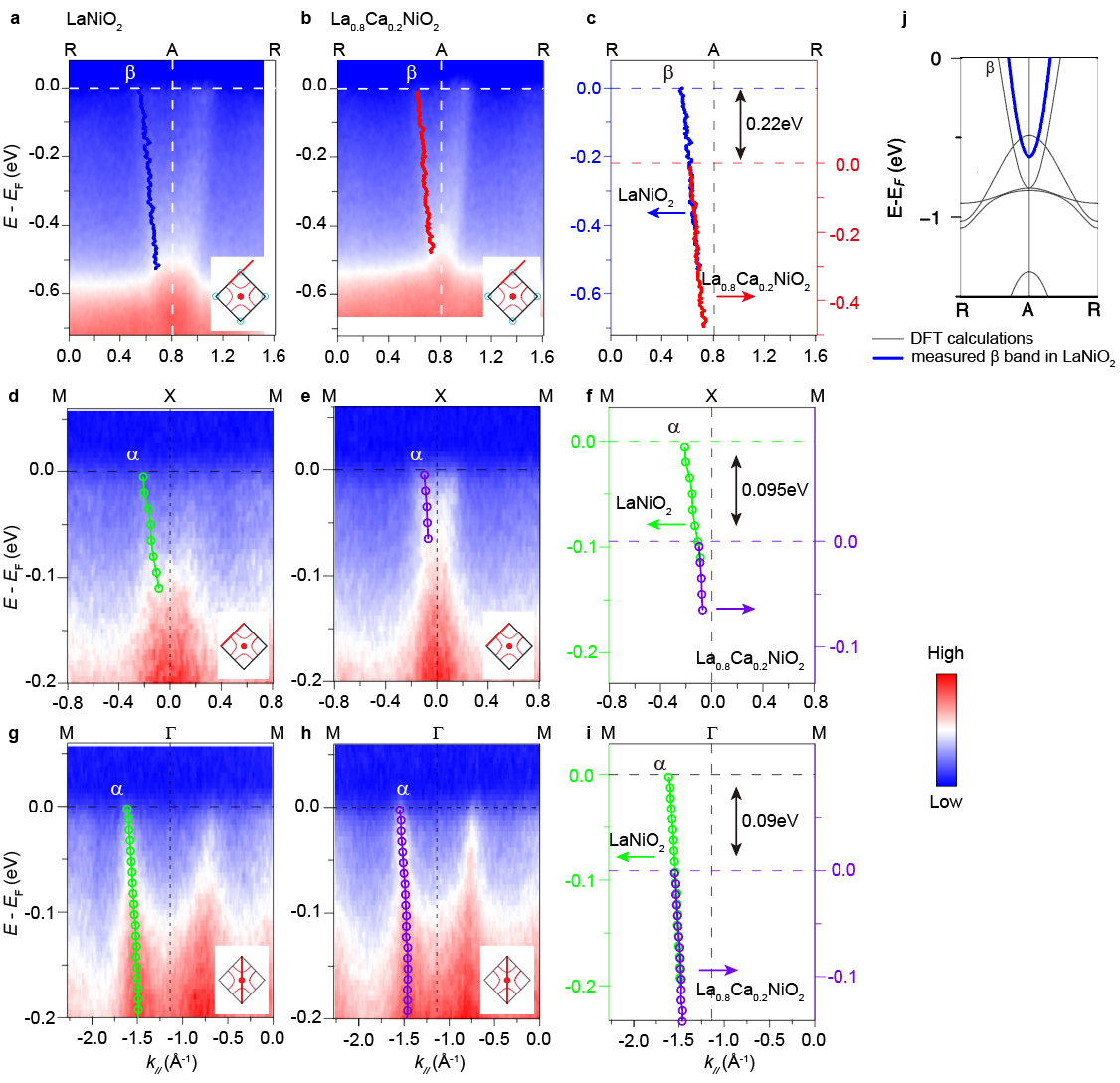


**Fig. S7.**

**Doping dependence of α and *β* bands between LaNiO_2_ and La_0_*_._*_8_Ca_0_*_._*_2_NiO_2_.** **a-b**, Photoemission intensity along R-A-R direction through *β* pocket of LaNiO_2_ (**a**) and La_0_*_._*_8_Ca_0_*_._*_2_NiO_2_ (**b**), respectively. **c**, Comparison of the dispersion of band *β* between LaNiO_2_ and La_0_*_._*_8_Ca_0_*_._*_2_NiO_2_. after an *E*_F_ shift of 0.22eV. **d-e**, Photoemission intensity along M-X-M direction through *α* pocket of LaNiO_2_ (**d**) and La_0_*_._*_8_Ca_0_*_._*_2_NiO_2_ (**e**), respectively. **f**, Comparison of the dispersion of *α* band along M-X-M between LaNiO_2_ and La_0_*_._*_8_Ca_0_*_._*_2_NiO_2_. after an *E*_F_ shift of 0.095eV. **g-h**, Same as (d-e) but along the M-Γ-M cut. **i**, Comparison of the dispersion of *α* band along the M-Γ-M cut between LaNiO_2_ and La_0_*_._*_8_Ca_0_*_._*_2_NiO_2_. after an *E*_F_ shift of 0.09eV. _._**j**, The dispersion of *β* band of LaNiO_2_ overlapping with the DFT calculation result. These indicate the non-rigid-band doping behavior in the IL nickelates.

_._


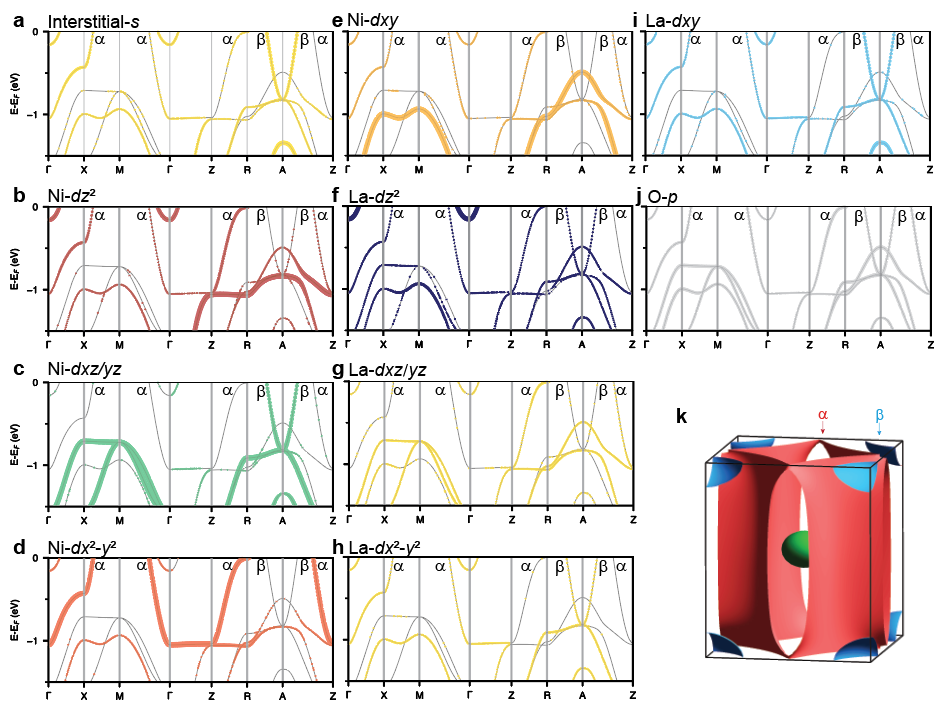


**Fig. S8.**

**Band structure from DFT calculation.** **a-j,** Projected orbital characters of the calculated band structure. The bands corresponding to *α* and *β* are indicated. **k,** Fermi surfaces of the calculated band structure.

**Table S1.**

The carrier density calculated from the experimental Fermi surfaces based on Luttinger theorem.

| Carriers/uc | *α*2*D* (holes) | *β*3*D* (electrons) | Total holes |
| --- | --- | --- | --- |
| LaNiO_2_ | 1.09 | 0.031 | 1.06 |
| La0*.*8Ca0*.*2NiO2 | 1.28 | 0.012 | 1.27 |
